# IDO and Kynurenine Metabolites in Peripheral and CNS Disorders

**DOI:** 10.3389/fimmu.2020.00388

**Published:** 2020-03-05

**Authors:** Yi-Shu Huang, Joy Ogbechi, Felix I. Clanchy, Richard O. Williams, Trevor W. Stone

**Affiliations:** The Kennedy Institute of Rheumatology, NDORMS, University of Oxford, Oxford, United Kingdom

**Keywords:** kynurenine, arthritis, lymphocyte antigens, 3-hydroxyanthranilic acid, Huntington's disease, T-cells

## Abstract

The importance of the kynurenine pathway in normal immune system function has led to an appreciation of its possible contribution to autoimmune disorders such as rheumatoid arthritis. Indoleamine-2,3-dioxygenase (IDO) activity exerts a protective function, limiting the severity of experimental arthritis, whereas deletion or inhibition exacerbates the symptoms. Other chronic disorder with an inflammatory component, such as atherosclerosis, are also suppressed by IDO activity. It is suggested that this overall anti-inflammatory activity is mediated by a change in the relative production or activity of Th17 and regulatory T cell populations. Kynurenines may play an anti-inflammatory role also in CNS disorders such as Huntington's disease, Alzheimer's disease and multiple sclerosis, in which signs of inflammation and neurodegeneration are involved. The possibility is discussed that in Huntington's disease kynurenines interact with other anti-inflammatory molecules such as Human Lymphocyte Antigen-G which may be relevant in other disorders. Kynurenine involvement may account for the protection afforded to animals with cerebral malaria and trypanosomiasis when they are treated with an inhibitor of kynurenine-3-monoxygenase (KMO). There is some evidence that changes in IL-10 may contribute to this protection and the relationship between kynurenines and IL-10 in arthritis and other inflammatory conditions should be explored. In addition, metabolites of kynurenine downstream of KMO, such as anthranilic acid and 3-hydroxy-anthranilic acid can influence inflammation, and the ratio of these compounds is a valuable biomarker of inflammatory status although the underlying molecular mechanisms of the changes require clarification. Hence it is essential that more effort be expended to identify their sites of action as potential targets for drug development. Finally, we discuss increasing awareness of the epigenetic regulation of IDO, for example by DNA methylation, a phenomenon which may explain differences between individuals in their susceptibility to arthritis and other inflammatory disorders.

## Introduction

**Figure 1:**
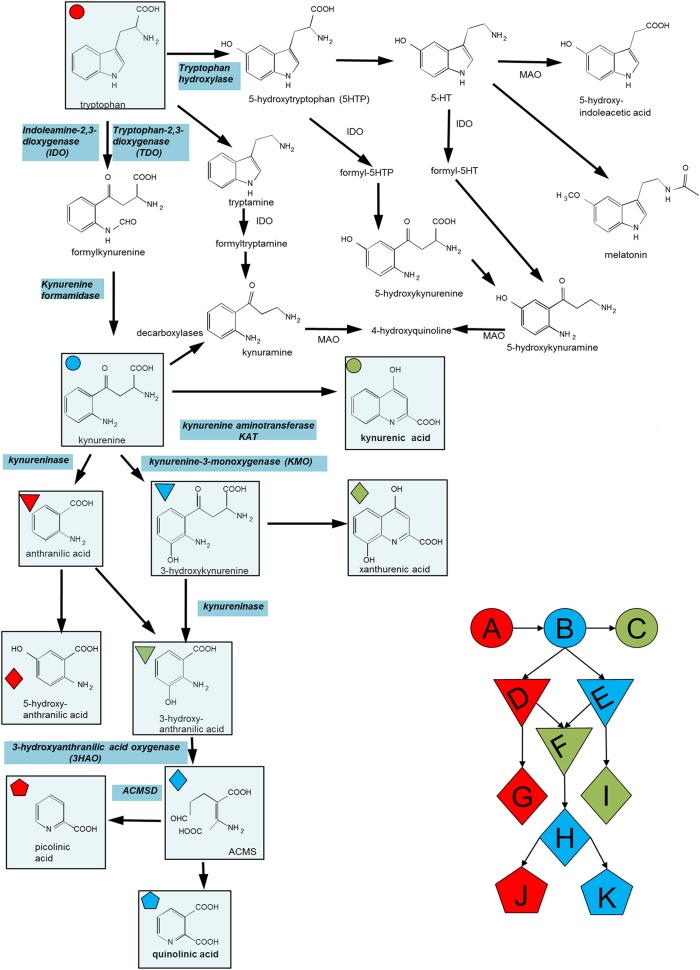
Summary of the major compounds and enzymes of the kynurenine pathway. ACMSD is α-amino-β-carboxymuconate semialdehyde decarboxylase. In cells lacking this enzyme the molecule in parentheses (ACMS) rearranges spontaneously (non-enzymatically) to quinolinic acid. When present, ACMSD converts ACMS to picolinic acid. Key metabolites include (A) Tryptophan, (B) Kynurenine, (C) Kynurenic acid, (D) Anthranilic acid, (E) 3-hydroxy-kynurenine, (F) 3-hydroxy-anthranilic acid, (G) 5-hydroxy-anthranilic acid, (H) ACMS, (I) xanthurenic acid, (J) picolinic acid, and (K) quinolinic acid.

**Figure 2:**
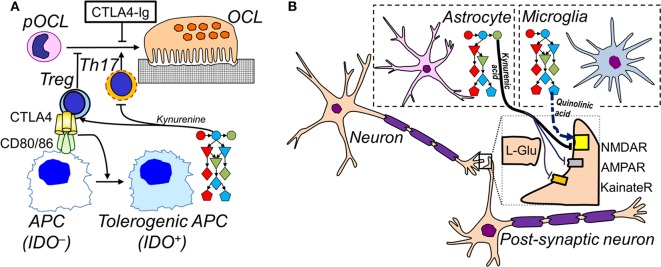
Cell-specific IDO pathways in inflammation**. (A)** In the immune system, APCs upregulate expression of the complete IDO pathway when activated. Tolerogenic APCs promote the differentiation of Tregs and inhibit Th17 differentiation. Tregs inhibit APC activation via CTLA-4, which also inhibits differentiation of pre-osteoclasts (pOCL) to osteoclasts (OCL); soluble CTLA4-Ig (ipilimumab) also inhibits OCL differentiation. Conversely, Th17 promote osteoclastogenesis. **(B)** In the CNS, microglia express low levels of kynurenine aminotransferase, pushing the IDO pathway to the production of excitatory and potentially neurotoxic quinolinic acid (dashed arrow). Astrocytes express low levels of KMO which leads to the accumulation of the NMDA receptor blocker and neuroprotective kynurenic acid (full arrows). See Figure 1 for legend to IDO pathway metabolites.

Widespread interest in the kynurenine pathway (Figure 1) and its roles in the nervous and immune systems developed in parallel from the discoveries of indoleamine-2,3-dioxygenase (IDO) activation by interferon-γ (1) and the subsequent discovery of a major functional role in placental immunity (2, 3) and the observation that catabolites of the IDO product, kynurenine, had modulatory effects on neuronal function (4–6) (Figure 2). It is now recognized that similar mechanisms may be involved at the molecular level of neuronal and non-neuronal processes and that activity along the kynurenine pathway is fundamental to the development of some central and peripheral disorders. Examples of these will be presented in this review, with the initial emphasis on disorders of primarily peripheral origin, including arthritis and atherosclerosis. A later section will emphasize the important links between peripheral and central inflammation by noting the roles of kynurenines and immune function in Alzheimer's disease, multiple sclerosis and Huntington's disease, where a relationship has been described between IDO and Human Lymphocyte Antigen-G. Those interactions may contribute to the roles of immune system activity in physiology and disease pathogenesis, with potentially common targets of therapeutic intervention for central and peripheral disorders.

## Kynurenines and Peripheral Inflammatory Disorders

### Rheumatoid Arthritis

The interface between tissue and immune system cells is seen clearly in peripheral inflammatory disorders such as rheumatoid arthritis (RA). In this condition, local tissue trauma and degeneration are accompanied by leukocyte infiltration and pannus formation which diminishes the volume of the joint space. This cellular infiltration and cytokine production eventually results in tissue damage and a positive feedback cycle in which the joint damage exacerbates the inflammatory response which in turn produces further bone and joint erosion.

Much work on the kynurenine metabolites in this condition has centered on the first enzymes of the pathway (IDO and tryptophan-2,3-dioxygenase, TDO), the isoforms of which have been discussed in depth (7). Where the identity of an isoform is known, it will be indicated in this review. The indeterminate form “IDO” implies that no distinction was made in the original literature. In general TDO, found mainly in the liver, exhibits greater selectivity for tryptophan whereas IDO, which occurs or can be induced in several tissues, acts on a wider range of indole-based substrates. As the enzyme most highly activated by interferon-γ (IFN-γ), IDO has often been the primary target used to explore disease mechanisms. In the case of RA, we have demonstrated that the inhibition of IDO (by 1-methyl-DL-tryptophan) or deletion of IDO1 increased the severity of arthritic symptoms in the collagen-induced model of arthritis (CIA) (8). The symptoms were, however, reduced significantly by the administration of kynurenine indicating that it was probably the loss of kynurenine or its downstream catabolites which were responsible for enhancing the arthritic symptoms and histopathology. Importantly, the effects of IDO inhibition included an increase in the numbers of IFN-γ- and interleukin-17- (IL-17)- producing T lymphocytes, particularly in the joints (8), suggesting a normally restraining influence of IDO. This is entirely consistent with the concept that RA is characterized by pathogenic T cells, including Th1 and Th17 cells.

In order to assess whether these observations might be relevant to RA in human patients, we considered mechanisms by which IDO or its activation processes might be affected in human subjects. One important mechanism for the regulation of IDO1 results from interaction between the B7 complex on dendritic cells (DCs) with Cytotoxic T-lymphocyte Antigen-4 (CTLA-4) expressed in the membranes of regulatory T cells. This ligation induces and activates IDO1 in the DCs and is maintained by Transforming Growth Factor-β (TGF-β) and inflammatory mediators via non-canonical actions of Nuclear Factor-κB (NFκB). This route is one of the major processes by which immune tolerance is maintained in a stable, long-term manner (9) and is an important link between arthritic damage and the kynurenine pathway. Subsequently we were able to demonstrate that in patients with RA there was a defect of IDO1 induction in the immune system involving the B7 / CTLA-4 interaction. The mechanism of this defect proved to be aberrant DNA methylation at the CTLA-4 promoter, leading to a loss of Treg cells and increased symptoms in the patients (10). The genetically impaired IDO1 activation thus reproduced the effect of arthritis exacerbation in IDO1–/– mice. The full cycle of events which explains the development, progression and remission of RA remains to be defined, but it is clear that IDO in DCs plays a significant role in that cycle. Since the simple procedure of administering kynurenine can reduce the degree of tissue damage and disability (8), this might represent a potential avenue for novel treatments.

It is perhaps unfortunate that IDO is such a clear and easily reproducible feature of inflammation since many studies have focussed almost exclusively on this enzyme and have interpreted the findings in terms of the full kynurenine pathway. The ratio of kynurenine to tryptophan concentrations in the blood or tissues has become a widely accepted marker of immune system activation, without a full appreciation of changes in the levels and relative amounts of metabolites downstream of kynurenine-3-mono-oxygenase (KMO). It is now clear that the effects of IDO activation are mediated not only via the reduced availability of tryptophan, but also by those downstream metabolites. These compounds have direct effects on the immune system that regulate the initiation, progression and termination of immune responses to infection or tissue damage.

Examples of these effects include the ability of kynurenine or kynurenic acid to activate the Aryl Hydrocarbon Receptor (AHR) which in turn induces an increased expression of IDO and TDO, providing a positive feedback circuit (11–14). The progressively increasing levels of kynurenine have two critical actions: induction of the transcription factor Forkhead Box-P3 (FoxP3) (15–17) and suppression of the transcription factor Retinoic Acid Receptor-related Orphan Receptor-γt (RORγt) (18, 19). FoxP3 promotes the differentiation of CD4+ T cells to CD4+CD25+FoxP3+ regulatory T helper cells (Tregs) which are able to inhibit other CD4+ cells and the cytotoxic effector T cells such as CD8+ T cells and Natural Killer (NK) cells. The inhibition of RORγt expression prevents the differentiation of CD4+ T cells into generally pro-inflammatory Th17 cells. Since the work described above had indicated a role for Th17 and Treg cells in CIA, involvement of the AHR and its feed-forward generation of IDO/TDO might be relevant.

Activation of AHRs by their classic agonist hydrocarbon molecules such as benzo[a]pyrene and 2, 3, 7, 8-tetrachlorodibenzo-p-dioxin (TCDD) could potentially account for the exacerbation of RA in cigarette smokers (20–22). Indeed, activation of AHRs by the constituents of cigarette smoke can potentiate the induction of NFkB by Tumor Necrosis Factor-α (TNF-α), leading not only to increased inflammatory activity but also reducing the efficacy of anti-TNF-α medications and possibly explaining both the apparent resistance of some patients to these drugs but also the high rate of non-compliance or discontinuation (23). The mechanism of AHR in these cases is likely to involve changes in the number and activity of Th17 cells (21) consistent with the evidence noted above. The exacerbation of symptoms in models of arthritis such as CIA and antigen-induced arthritis (AIA) is prevented by deletion of either AHRs or of IL-17 receptors (21) supporting the view that AHR activation can drive Th17 differentiation.

Helping to understand the mechanisms underlying RA, osteoarthritis (OA) and osteoporosis (OP) is the finding that the effects of AHR activation are potentiated by Human Leucocyte Antigen-DRB1, a significant risk factor for the development of arthritis (24). The synergism is sufficient to increase osteoclast generation and thus bone damage but, even more relevant systemically, the combination increases Th17 cell differentiation with high IL-17 levels noted in arthritic joints and draining lymph nodes.

Conversely the activation of AHRs by compounds with known anti-arthritic potential, such as sinomenine and norisoboldine, promote the differentiation of Treg cells (25, 26). Since the AHR is known to respond differentially to various compounds and to produce effects that depend qualitatively on the agonist, it will be of interest to determine whether these effects reflect a known or novel target site on the AHR complex which might be amenable to new drug development. An unrelated compound, tetrandrine, combines both these actions and normalizes the Th17: Treg ratio by a mechanism which involved the AHR (27). However, it is unclear whether the compound acts on two distinct target sites on the AHR, or whether the result of acting at a single site drives two different responses in Th17/Treg precursor cells. It is possible, for example, that tetrandrine has different sites of action in the two populations or that it induces different responses when acting on AHRs in cells destined to become Th17 and Treg precursors. These uncertainties would be important matters to clarify as they could lead to more selective molecules with a single mechanism of action which might be devoid of unwanted effects on other cell populations.

In addition to these promoters of AHR activity, several routes are now known by which AHR activity can be down-regulated. The microRNA molecule miR-223 for example suppresses AHR activity by interfering with the AHR Nuclear Translocator (ARNT) (28). Of particular interest was the observation that this interaction occurred in macrophages, but was less prominent in patients with RA than those with osteoarthritis, supporting proposals that these cells play an important role in the etiology of RA. However, these results are difficult to reconcile with the report that AHR deletion from thymocytes reduced arthritic parameters in mice with CIA, whereas deletion selectively from macrophages had no effect (13).

Since AHR expression is seen in most cell types, it is important to consider that immune system cells may be the dominant population involved in the regulation of arthritic inflammation. Nevertheless, it has been reported that the fibroblast-like synovial cells, which exhibit a high rate of proliferation and migration within the joints of patients with RA, also express AHRs. Activation of the receptors comprehensively inhibits the synovial cells, reducing proliferation, migration and invasion of surrounding tissue (29). A role for IDO in these phenomena has not been studied in depth but would be predicted as a significant feature of AHR activation elsewhere.

Although there is continuing controversy about the relative importance of tryptophan depletion and kynurenine metabolite activity in immune system regulation (30, 31), it is likely that both mechanisms are relevant to some extent. While kynurenine and its metabolites are the primary biologically active compounds in the kynurenine pathway, the changes of their concentrations in blood or CSF are often small, as is the reduction in tryptophan concentration resulting from its oxidation (to kynurenine) by IDO or TDO. Much of the behavioral work in experimental animals, or clinical work in humans, includes the measurement of the kynurenine: tryptophan (K/T) ratio, which provides a larger and statistically more useful parameter. The effects of tryptophan depletion are likely to be mediated by activation of the Generalized Controller Non-derepressible-2 Kinase (GCN2) (16, 32, 33), a sensor of cellular amino acid levels. A reduction in the concentration of tryptophan, for example, results in increased numbers of free tryptophanyl-tRNA molecules which induce GCN2 activation. Cells expressing IDO, such as plasmacytoid DCs, therefore, can induce T cell anergy by reduced proliferation and induced apoptosis (32, 34). Although GCN2 is usually considered to be necessary for linking tryptophan deficiency to the inhibition of cell cycling, it is possible that this relationship operates differently in CD4+ and CD8+ T cells (35). The effect of tryptophan depletion is potentiated by tryptophan catabolites in the kynurenine pathway (16).

### Osteoporosis

Osteoporosis is another musculo-skeletal disorder in which inflammation is thought to play a significant role although the involvement of kynurenines remains poorly understood. When quantifying the levels of kynurenines in patients newly diagnosed with osteoporosis (and therefore receiving no relevant medication at the time), it was found that the serum content of 3-hydroxy-anthranilic acid (3HAA) was substantially *lower* (around 10-fold) than that in a parallel cohort of normal control subjects. Moreover, there was a comparably *increased* concentration of anthranilic acid (AA), generating an overall difference in concentration between these compounds of approximately 100:1. After treatment with the standard drugs - bisphosphonates or Selective Estrogen Receptor Modulators (SERMs) for 2 years, these values had returned to the levels determined in control subjects, accompanied by a significant improvement in measurements of bone density (36).

Both the mechanism and the pathological significance of this remain unclear. There have been suggestions of enzymic conversion of AA to 3HAA (37) which, potentially, might be inhibited during the course of inflammation resulting in a higher AA: 3HAA ratio. It is not known, however, whether these changes in the kynurenine pathway are primary or secondary contributory factors in the development of osteoporosis. 3HAA exerts inhibitory control of Th1 cells, changing the important ratio between inflammatory, IFN-γ-secreting Th1 cells and anti-inflammatory IL-10 secreting Th2 cells with a resulting anti-inflammatory polarization of immune system function (38). A loss of 3HAA should therefore result in a pro-inflammatory environment and could account for the emergence of a disorder such as osteoporosis in which the immune system is likely to be involved (39–41).

But what could generate the loss of 3HAA? Could it simply be a defective enzyme converting anthranilate to AA to 3HAA? Or might there be a reduced oxidation by KMO of kynurenine to 3-hydroxy-kynurenine (3HK), with kynureninase catabolising the excess kynurenine to AA? Why then is there no comparable increase in the conversion of kynurenine to kynurenic acid via kynurenine aminotransferase (KAT)? Do therapeutic agents affect the kynurenine pathway directly, in which case those effects might drive the AA:3HAA ratio and determine the initiation and time course of inflammation, or are all the kynurenine pathway changes a result of altered levels of a crucial factor such as a regulatory cytokine, chemokine or growth factor? Certainly 3HAA is less stable than AA in aqueous media, as discussed previously (42), largely because it is a reactive compound which auto-oxidizes to a form which dimerises spontaneously to cinnabarinic acid (43, 44). However, this molecular difference does not readily account for the differences in concentrations observed between two populations of patients, since the chemical and redox environment should not differ greatly between the groups.

The importance of these questions lies not simply in an understanding of osteoporosis, but also in accounting for similar changes in a wide range of disorders in which an underlying inflammation appears to be involved. Thus, similar, though not as dramatic, changes in AA: 3HAA ratio have been reported in a range of disorders [see (30, 31, 42)] where they can change progressively with the development of disease symptoms (45).

In attempting to explain some of these phenomena, several groups have turned to vitamins and the possible relevance of a deficiency in their availability. One plausible view is that disturbances to the kynurenine pathway may result from a deficiency of pyridoxal phosphate, one form of vitamin B6 which is a crucial co-factor for several kynurenine pathway enzymes such as kynureninase and kynurenine aminotransferase. Although not previously considered, riboflavin (vitamin B2) is also required, along with pyridoxal phosphate, for the activation of KMO (46, 47). Infections and inflammation are associated with increased turnover of this cofactor (48), lowering cytosolic concentrations, a relationship consistent with the ability of riboflavin to inhibit the production of inflammatory cytokines (49), to potentiate the effects of anti-inflammatory drugs (49, 50) and to enhance host resistance (51, 52). Conversely, reduced riboflavin availability is associated with greater inflammatory activity in arthritis (53). Thus, the lowering of riboflavin content associated with the onset of inflammation, in turn producing suppressed KMO activity and the preferential metabolism of kynurenine to AA, could account for the high AA: 3HAA ratio observed in osteoporosis (36). As noted above, a similar elevation in this ratio has been reported in patients with arthritis (54) and other chronic disorders involving tissue inflammation (42).

As in RA, the AHRs seem to play a significant role in the regulation of bone turnover and fragility. Activation of AHRs enhances osteoblast production with corresponding increases in bone formation and strength (55). Similarly, Michalowska et al. (56) reported that kynurenine promotes osteoblast formation, possibly resulting from its inhibition of myeloid mesenchymal stem cell proliferation and the consequent loss of osteoblast precursors. On the other hand, there is evidence that the renal deficiency which results from kidney damage or loss increases the expression of AHRs in osteocytes and elevates plasma levels of a range of metabolites including kynurenine and 3-hydroxykynurenine (3HK) (57). These changes are associated with increased numbers of osteoclasts, with kynurenine levels inversely related to several parameters of bone formation. Overall it was suggested that the AHR-mediated production of kynurenines mediated the osteoclast generation. In addition, while AHR generation of kynurenines promotes bone formation, the activation by AHR of the Cytochrome P450 enzymes promotes osteoclast activity so reducing osteogenesis, whereas AHR deletion was reported to increase bone development and density (58). It is clearly important not only to establish the reasons for the differing conclusions on kynurenine activity but also whether the various effects of AHR activation are mediated by kynurenine metabolites, cytochrome enzymes or other routes, and how those different routes interact with each other and with overall tissue function.

Also introducing an element of confusion is evidence that the presence of inflammation may alter the actions of AHRs. Thus, increased expression of AHRs was demonstrated in mesenchymal stem cells from mice with CIA (59) and this was associated with reduced production of osteoblasts. Treating the animals with AHR agonists further reduced bone formation, potentially representing a contributory mechanism in osteoporosis (55). Clearly it is essential to know whether the behavior of cells and receptors differs significantly between a normal physiological state and that of the pathological, diseased state since it would impact on the approach needed for therapeutic developments.

These various factors may also be complicated by dietary considerations. Increasing attention is being devoted to dietary regulation of metabolism, with directly acting AHR agonists such as 3,3′-diindolylmethane being of special interest. This and related compounds occur in several *Brassica* species of vegetables and have been found to inhibit osteoclast numbers and activity, resulting in enhanced bone formation (60). Again, it will be valuable to establish whether the kynurenine pathway is involved in this, and how it interacts with, and possibly modifies, the other consequences of AHR activation.

The importance of kynurenines on bone formation is dependent not only on AHR expression but also on activity in the CTLA-4 and B7-mediated interactions between T cells and DCs. CTLA-4 inhibits T cell activation by blocking the interaction between the T cell receptor co-activator CD28 and the B7 (CD80/CD86) complex. This results in increased generation of Wnt-10b which promotes bone formation (61).

The activation of IDO in DCs is also regulated by the B7 complex, with activation by CTLA-4 expressed on Tregs producing a tolerogenic profile in the DCs. The loss of bone tissue which follows the menopause or ovariectomy has been associated with increased activity in DCs, but preventing CTLA-4 activity preserves bone tissue (62), suggesting a potential use of the stable, synthetic construct CTLA-4Ig in reducing osteoporosis. As with several other instances of IDO and kynurenine involvement in pathology, it remains uncertain whether the modulation of DC activity is via local changes in tryptophan concentration or the generation of pro-apoptotic compounds such as 3HK and 3HAA.

In addition, CTLA-4 acts directly on the CD80/86 proteins on monocytes to inhibit their differentiation to osteoclasts, resulting in reduced bone destruction (63–66). Clearly, with such a range of sites of action for CTLA-4 and CTLA-4Ig, some of which influence bone formation and destruction, it is uncertain how important the regulation of kynurenine and its catabolites is to the overall control of bone formation in health or disease. In particular, it is not known whether changes in the concentration of any components of the kynurenine pathway are able to modulate any of the interactions between CD28, CD80, CD86, and CTLA-4. In view of the growing use of CTLA-4Ig in RA, and of the CTLA-4 blocking antibody ipilimumab in melanomas, however, these questions might be worthy of investigation. It is tempting to speculate on the range of studies that, while not specifically examining the actions of kynurenine and its metabolites, nonetheless have generated results which might contribute to understanding the full extent of tryptophan metabolite involvement. Thus, while the anabolic steroid dehydroepiandrosterone increased osteoblastogenesis and bone formation, it also increased the numbers of FoxP3+ Treg cells, an effect that might be generated via kynurenine pathway activation (67).

Finally it is interesting to note that bone formation may be affected indirectly by changes in vitamin D metabolism. Benzo[a]pyrene, for example promotes the catabolism of the vitamin and would thus hinder calcium absorption and bone formation (68). This would certainly be an important consideration for individuals concerned about osteoporosis following many years of cigarette use. It would be of great interest to determine whether these effects are the result of benzo[a]pyrene activation of AHRs and the subsequent activation of IDO or TDO.

### Atherosclerosis

The overall anti-inflammatory effect of kynurenine in arthritis is reflected in similar properties in several other peripheral disorders. Atherosclerosis is characterized by vascular endothelial deposits known as plaques in which accumulated leukocytes are tightly enmeshed with a calcified complex of fatty materials, blood cells and platelets. These plaques reduce the effective diameter of the vascular lumen, restricting blood flow and affecting blood pressure and tissue viability as a result (69). Atherosclerosis is a major risk factor for disability and mortality and a full understanding of the underlying causative factors remains uncertain. Although the regulation of cholesterol metabolism by apoenzyme-E (ApoE) is believed to play a prominent role in the disorder, rodents specifically lacking ApoE exhibit little vascular abnormality. When ApoE deficiency is combined with the deletion or inhibition of IDO1, however, there is a marked exacerbation of the pathology, as noted above in arthritis, with the deposition of atherosclerotic plaques comparable to the natural disorder in humans (69, 70). The importance of IDO activation has been demonstrated by reports that the promotion of Treg differentiation by IDO-expression tolerogenic DCs produces a reduction in atherosclerotic plaque deposition (71, 72).

Consistent with the studies on trypanosomiasis described below (section Psychiatric Disorders), in which kynurenine levels were positively correlated with IL-10, down-regulation of IDO1 in the atherosclerosis model resulted in a diminished expression of IL-10 in most lymphoid tissues including peripheral blood, spleen and lymph node B cells (69). However, there is not a simple cause-and-effect relationship between these compounds since IL-10 expression was not induced or elevated by kynurenine administration *in vivo*. On the other hand, kynurenine did increase IL-10 production by B cells *in vitro* (69).

Not only are these results interesting in terms of understanding the importance of IDO activity in maintaining immune tolerance and thus restraining the extent of disease, but they support the possible explanation of some apparently conflicting results noted above. If the kynurenine induction or promotion of IL-10 expression is specifically exerted on B cells, the overall relationship between the two compounds will depend on the relative involvement of T cells, B cells and probably other leukocyte populations, as stated earlier.

## Central Neuro-Inflammation

Understanding the role of kynurenines in the Central Nervous System (CNS) has involved work in two areas of tissue function which are more problematic in this region than elsewhere. The first is the question of pathway localization at the cellular level. Detailed histological studies on the presence and distribution of the various enzymes along the kynurenine pathway revealed a differential localization in cell types. For example, KMO was absent from astrocytes (73, 74), so that these cells are only able to generate the glutamate antagonist and neuroprotective kynurenic acid. In contrast, microglia express all components of the pathway, so that their activation by inflammatory mediators could result in increased levels of quinolinic acid. This is consistent with the phenotypic resemblance of microglial cells to that of resident phagocytes in other organs, as macrophages also convert tryptophan to all components of the kynurenine pathway including quinolinic acid (74–76). The ability to generate quinolinic acid and other damaging kynurenines such as 3HK, explains why the inflammatory activation of microglia could cause local cellular damage or death. As IDO1 was constitutively active in most glial cells (74, 77), it had not been clear why the generation of quinolinic acid would not produce a significant ongoing loss of cells. That could now be understood as the result of kynurenine and kynurenic acid production by astrocytes which—in the absence of KMO and downstream enzymes—could accumulate these compounds to a level at which they could antagonize glutamate and quinolinic acid to prevent neural over-excitation and excitotoxicity (73, 77). Kynurenine crosses cell membranes readily and kynurenic acid, despite its poor membrane permeability, also enters the extracellular space (78, 79). Whether this is simply the result of slow diffusion, a facilitated transfer by pinocytosis or the excretion of kynurenate in exosomes, remains unclear.

There remains significant debate on this hypothesis, as quinolinic acid synthesis by activated microglia may exceed kynurenate production, resulting in local cell damage. In such an inflammatory situation, IDO will also be activated in astrocytes and the increased production of kynurenine—in view of its higher membrane permeability—could well diffuse into the activated microglia much more rapidly than would kynurenic acid. The result could be a potentiation of microglial kynurenine metabolism with even greater levels of quinolinic acid being produced.

Interestingly, the expression of KAT can also vary between cell types in the CNS. Microglia express lower levels of the more active KAT2 relative to KAT1 (73) so that the conversion of kynurenine to quinolinic acid in these cells will be further enhanced relative to cells expressing more of this enzyme. Subtle differences such as these between cell types may be critical in regulating the relative production of different kynurenine metabolites depending on the balance of neural and glial cell activity under any given functional circumstances such as different cytokine profiles.

### Huntington's Disease

The term “central neuro-inflammation” encompasses several disorders in which the initiating defect involves neurons or glial cells but which results in abnormal functioning of the nervous system as a whole. One such disorder is Huntington's disease which can now be understood in terms of newly recognized interactions between kynurenines and aspects of immune function and which may contribute to their roles in physiology and disease pathogenesis.

In the CNS many of the pathological effects of kynurenine pathway activity may result from a balance between the concentrations of quinolinic acid, an agonist at glutamate receptors sensitive to N-methyl-D-aspartate (NMDA) (4, 6, 80) and kynurenic acid, an antagonist at glutamate receptors but with greatest efficacy blocking NMDA receptors (5, 81, 82). An excess of the former may result in excessive depolarization, calcium influx and neurotoxicity (83), leading to the speculation that an over-production or suppressed removal of quinolinate might be a factor in neurodegenerative disorders such as Huntington's disease with a possibly similar role in Alzheimer's disease and other central disorders (84, 85).

In addition, over-activation of NMDA receptors is accompanied by increased microglial proliferation and activation together with cytokine production. This indicates that an inflammatory environment has been induced in which the levels of potentially pathogenic cytokines are likely to cause, or contribute to the induction of neuronal damage or death (86, 87). Consistent with this idea, the levels of quinolinic acid in the CNS are increased by at least two orders of magnitude during and following viral infection (88, 89), or following exposure of experimental animals to Toll-Like Receptor agonists such as bacterial lipopolysaccharides (LPS) or viral-RNA-mimetic poly(I:C). These phenomena recall the induction and activation of the kynurenine pathway by IFN-γ (1) and the importance of these phenomena in host responses to infection, the protection of allogeneic embryos or the maintenance of tissue grafts resulting from the depletion of tryptophan and generation of kynurenine catabolites (2, 3, 90–92).

It is now clear that there are also major changes in the immune system associated with some central disorders, of which Huntington's disease represents a good example. Huntington's disease is a genetic, inherited disorder in which the early degeneration of striatal regions produces progressive motor disorders and later cortical involvement leading to cognitive dysfunction. It is one of few conditions in which involvement of the kynurenine pathway has been implicated on the basis of studies on prokaryotes, *in vitro* and *in vivo* mammalian models and human patients (93–95) and for which the kynurenine pathway has been considered as a therapeutic target (96–98). Increasingly, evidence indicates parallel disturbances in the immune system (94, 99–102). Central microglia are activated and peripheral monocytes of patients with Huntington's disease are hyper-sensitive to immunogenic stimuli (103). A variety of changes in cytokine expression have been reported in the disorder (100, 104) and it is claimed that immunosuppressant treatment can produce significant amelioration of Huntington's disease symptoms and progression (105).

Particularly intriguing is the finding that soluble Human Leucocyte Antigen-G (sHLA-G) levels in the serum of patients show a clear trend of correlation with symptom severity, and a statistically significant effect in the most severely affected patients (100). HLA-G has been associated primarily with materno-fetal tolerance (106) but elevated HLA-G expression has been noted in several CNS disorders in which its effects are largely immune-suppressant (107, 108). The full importance of this was not appreciated at the time of the original study, but the molecule is of great interest since it is a secreted protein with the ability to inhibit T cell and cytotoxic cell activity (109).

sHLA-G also promotes the differentiation of Treg cells (110), the effect of which is to enhance the progression and metastasis of tumors. IDO expression is greater in tumors than normal tissue so that these two molecules—IDO and sHLA-G—might act synergistically as tumor promoters. Such synergism might explain the poor performance of selective IDO1 inhibitors in clinical oncogenic trials (111–113). Since IDO1 and sHLA-G are not restricted to the cell membrane or cytoplasm, but can be released into the extracellular medium, their immunosuppressant activity will be exerted around a wider cellular environment than would otherwise be the case with corresponding implications for generalized inflammation and tumor surveillance.

Importantly, it has been proposed that under some circumstances, especially in the presence of high concentrations of IFN-γ, the major immunosuppressive activity of activated DCs may be mediated to a greater extent by the expression and release of sHLA-G than by IDO (114). The inhibition of cytotoxic CD8+ T cells is largely prevented by antibody blockade of sHLA-G rather than by IDO inhibition. The relationship between high levels of IFN-γ and HLA-G is quite specific since antigen induction is around 20-fold greater than other HLA antigens and neither IL-6 nor IL-10 mediate the effects of interferons on HLA-G expression. It has been suggested that the relative potencies of IDO and HLA-G-mediated immunosuppression might contribute to the dual nature of interferon activity, this being a major Th1 cytokine inducing inflammatory mediators in the early phase of immune responses to stimuli, but exerting an auto-limiting suppression of inflammation in the later phases (115). High concentrations of IFN-γ induce HLA-G expression in DCs which are then responsible for cytotoxic T-lymphocyte inhibition. HLA-G also acts on macrophages (116) to promote their differentiation into the M2 phenotype which is characterized by increased CD163 and reduced CD86 expression. Placental and decidual M2 cells are involved in fetal protection against maternal T cell attack *in utero* (117–119) with increased activity of IDO1, a known contributor to fetal protection. Overall, therefore, the parallel changes in Huntington's disease severity and sHLA-G expression and their correlation with the genetic mutation (100) may reflect a significant relevance of immune function in the progression of the disorder.

Questions also remain about these relationships, especially that between immune system function in the peripheral and CNS, given that markers of inflammation have been widely reported in the blood and peripheral organs and tissues of patients with Huntington's disease. A version of the mutated huntingtin protein—which is believed to the primary cause of neurodegeneration in Huntington's disease—also induces disturbances to immune function (99) and there are marked similarities in the altered profile of gene expression in the blood cells and neurons of Huntington's disease patients (104). Do these changes arise simultaneously from an undefined factor, or does one act as a trigger for changes in the other? What would happen in patients treated with an anti-inflammatory agent which was confined to the blood and peripheral tissues? Would such a drug break the degeneration-inflammation cycle sufficiently to reduce the symptoms of Huntington's disease or, possibly, halt its progression? Highly relevant to these considerations are reports that the treatment of patients with Huntington's disease using an immune-modulating drug such as glatiramer acetate showed significant beneficial effects (105), emphasizing the need to understand the neuro-immune interactions at the heart of the disorder.

### Alzheimer's Disease and Multiple Sclerosis

In addition to its activation of NMDA receptors, quinolinic can induce the expression of immunologically active molecules in astrocytes including IL-8, Chemokine Ligand-5 (CCL5) and Macrophage Inflammatory Protein-1 (MIP-1) as well as several chemokine receptors such as CXCR4, CXCR6, CCR3, and CCR5 which promote leukocyte attraction across the blood-brain barrier (120, 121). Several of these proteins are increased in the brains of patients with Alzheimer's disease, so the similarity of this activity to the action of β-amyloid fragments has prompted the suggestion that the kynurenine pathway may be involved in the etiology of this disorder, an idea consistent with the demonstration that amyloid-β induces IDO expression (122, 123). It is possible that quinolinic acid could initiate a positive feedback cycle of cell activation, further quinolinic acid generation, mediator induction and further activation, from which it may be difficult for cells to escape. Stimulation of NMDA receptors by quinolinic acid stimulates glial proliferation, further enhancing these events (120, 124).

The increased kynurenine pathway activity in Alzheimer's disease (74, 77, 125, 126) has been linked with β-amyloid production and tau hyperphosphorylation which can be induced by quinolinic acid (74, 77, 122, 124, 127, 128). There is also good evidence that the kynurenines plays a role in the formation of neurofibrillary tangles and senile plaques (126). IDO1 inhibition suppresses plaque development, neuronal death and cognitive dysfunction (60).

Neurons express ACMSD (Figure 1) and can therefore divert the conversion of 3HAA from quinolinic acid to picolinic which also regulates inflammatory mediator release (129–131). It also prevents some of the deleterious actions of quinolinic acid including its toxicity on cholinergic and dopaminergic neurons (132–134). It has been suggested that this component of the kynurenine pathway is important in the production of suicidal ideation and behavior (135).

Some of these considerations are especially important in multiple sclerosis (MS) where the underlying problem is inflammatory and the products of kynurenine pathway activation, primarily quinolinic acid, are toxic not only to neurons but also to oligodendrocytes, thus contributing to the loss of central myelination. Kynurenine pathway activity is abnormal in patients with MS, suggesting cell activation (136–139). In patients with MS or in the animal model of this disorder (experimental autoimmune encephalomyelitis, EAE), IDO1 and KMO are increased (85, 137, 140) possibly as a result of the high levels of TNF-α and IFN-γ. IDO inhibition exacerbates EAE severity in mice (141, 142). MS appears to be a disorder in which the beneficial effects of kynurenine pathway activation (induction of T cell tolerance) is in competition with the generation of potentially damaging levels of quinolinic acid (76, 143).

The question of membrane permeability by kynurenine is also important in the relationship between kynurenine pathway activity in the peripheral circulation or tissues and the CNS. The stimulation of immune system cells activates several enzymes in the kynurenine pathway which are relevant to the control of leukocyte populations and the balance between pro- and anti-inflammatory cells and their products. At least two of these—quinolinic acid and kynurenic acid, as noted above—can regulate neuronal excitability and plasticity. It could be life-threatening if the delicate CNS control of bodily functions—somatic and autonomic—were to be influenced significantly by the variety of infective, traumatic, allergic or inflammatory changes that involve the peripheral immune system. The brain is protected from these, however, by the blood-brain barrier, across which kynurenine and 3HK can cross quite readily, but quinolinic acid and kynurenic acid cross very slowly. Indeed, the clinical consequences of intense or chronically elevated levels of peripheral immune system activation appears to be a major factor in several psychiatric disorders such as depression (144) and schizophrenia (145–147) resulting from the altered balance between kynurenic acid and glutamate receptor agonists, including quinolinic acid. Some of these issues have been discussed in detail elsewhere (148). It has been shown that the cerebrovascular cells intimately involved in blood-brain barrier function express elements of the kynurenine pathway. On activation, the vascular endothelial cells and pericytes produce kynurenine which is released from basolateral sites providing a short path to diffuse across into the brain parenchyma. They also synthesize kynurenic acid which, with picolinic acid, are protective by their abilities to block glutamate receptors and to suppress the secretion of inflammatory mediators, respectively. It has been pointed out that these properties of the blood-brain barrier may be critical factors in HIV-Associated Neurocognitive Disorder (HAND) since the kynurenine produced from barrier cells by systemic inflammatory mediators can enter the CNS rapidly in large amounts, being then converted by microglia to quinolinic acid at a rate sufficient to overwhelm kynurenate production or entry. The resulting excitotoxic loss of neurons may than contribute significantly to the emergence of dementia (148).

Quite apart from the neuroimmunological activity of quinolinic acid, it can increase the permeability of the blood-brain barrier (149–152), an effect intriguingly opposed by kynurenic acid (153). Not only would this allow inflammatory mediators easier access to the CNS cells but it would also facilitate passage of leukocytes directly into the brain parenchyma.

### Psychiatric Disorders

The kynurenine pathway may be involved in several psychiatric disorders, several of which have been reviewed in detail (144–146, 154–157). Perhaps the strongest evidence is for a role in schizophrenia (145, 146). One issue which has aroused controversy in this area is a claim that the role of kynurenic acid in schizophrenia could involve the block of nicotinic receptors in addition to NMDA receptors. While the levels of kynurenic acid are increased in the CNS and probably contribute to the symptoms of schizophrenia (146) and other CNS disorders involving defective cognition, that claim has not been substantiated and cannot be replicated [see (158)]. Any apparent effect of kynurenic acid on nicotinic receptors appears to be secondary to the effects of nicotinic receptors on the release of glutamate and other neuroactive compounds (158).

There is also very good evidence that activation of the kynurenine pathway is a major factor in the production of depression and related illnesses. Certainly the K/T ratio correlates well with the induction and severity of depressive symptoms following the administration of IDO inducers such as interferon-β or exposure to stress (144, 154–157). The levels of kynurenines are also closely associated with severe depression and the development of suicidal thoughts and behavior (135).

There has been less interest in the role of kynurenines in anxiety disorders, probably because of the difficulties of interpretation in studies of a psychological process which is hard to translate from experimental animals to humans. Nevertheless, there is increasing evidence that the kynurenine pathway is involved in anxiety behaviors, especially related to primary events in the immune system, such as inflammation (159).

### Malaria and Trypanosomiasis

Infective, rather than genetic examples of neuroinflammation are the parasitic infestations of cerebral malaria and trypanosomiasis. Approximately 20% of people who contract malaria will proceed to develop cerebral malaria, a condition which causes serious somatic and psychiatric changes in patients and, in most cases, results in death. Infection of C57BL/6J mice with the strain *Plasmodium berghei ANKA* generates an animal model in which the cerebrovascular histology and functional involvement closely reproduce these phenomena in humans (160). When treated with *P. berghei ANKA* almost all the tested mice died within 7 days, but animals treated simultaneously with Ro61-8048 (an inhibitor of KMO) survived up to the end of the study at 21 days (160). KMO inhibition increased the endogenous levels of kynurenine and kynurenic acid as reported in other studies (161–164) and seen in KMO-deficient animals (165, 166). The blockade of neuronal glutamate receptors by kynurenate was probably the main factor accounting for the protective inhibition of neurotoxicity and animal survival. Although this initial study did not take account of changes in the immune system, the results were sufficiently clear that a similar study was performed subsequently in trypanosomiasis. Here, an animal model was used to assess the infiltration of brain parenchyma and vascular or ventricular endothelia by reactive leukocytes (167). The results indicated that KMO inhibition was able to reduce significantly the extent of leukocyte infiltration into the brain as well as the histological assessment of neuronal death.

To follow up these results in human patients, levels of kynurenine and its catabolites were measured in the cerebrospinal fluid of patients suffering from trypanosomiasis and the samples were also examined for levels of IL-6 and IL-10. There was a close and highly significant correlation between levels of kynurenine and IL-6, supporting the view that an inflammatory response had been initiated within the CNS. However, there was also a strongly positive correlation between kynurenine and IL-10, a largely immunosuppressive cytokine secreted primarily by anti-inflammatory leukocytes which suppress DC activation and IDO expression. It is not obvious why there should be an increase in IL-10 production associated with tryptophan metabolism, but several other groups have also reported positive relationships between the kynurenine pathway and IL-10 (168–171). In a cohort of healthy young individuals a clear positive association was seen between IL-10 levels and those of kynurenine, the kynurenine: tryptophan ratio, 3HK and 3HAA levels (172). A reduction of TNF-α and IL-17 expression by mesenchymal stem cells has been associated with increased IL-10 and kynurenine levels (170), although LPS produces parallel increases in TNF-α, IL-10, and IDO activity (173).

In other cases kynurenine and IL-10 behave differently. Subjects responding to BCG vaccinations and patients with inflammatory bowel disease have increased kynurenine and IFN-γ levels but reduced IL-10 as expected of an immune response (174, 175). IDO1 activity has been shown to increase IL-10 production in B cells, whereas *in vitro* kynurenine did not do so, implying that another IDO1 product might be involved. A correlation was reported between pro-inflammatory TNF-α and kynurenine levels, whereas the neuroprotective kynurenic acid was correlated with IL-10 levels (176). Indeed kynurenic acid has been reported to increase IL-10 production (177) so whether kynurenine production is positively or negatively related to IL-10 may depend on the balance of B cell, T cell and monocyte activity in addition to being dependent on activity in different parts of the kynurenine pathway (Figures 1, 2).

Administration of the statin group of drugs reduces IL-6 production but increases IL-10 and kynurenine (178). This expected opposite movement of IL-10 and IL-6 has been observed in IDO1-deficient DCs in which there is the anticipated loss of AHR activity and reduced levels of IL-10, but increased IL-6 and TNF-α production (179); conversely both IL-6 and IL-10 were increased after surgery (180). Kynurenine itself suppresses IL-6 release but kynurenic acid has been reported to increase it. Since both compounds increase IL-10 production, the ratio between kynurenine and kynurenic acid may be a particularly important factor in determining the inflammatory cytokine balance (181).

Nevertheless, despite their opposite immune system bias, IL-6 and IL-10 have been shown to change in parallel in patients with depression, in which inflammatory drive (IFN-γ, TNF-α, CRP) is reduced. In this same condition IDO activation increases kynurenine concentrations, producing a negative correlation with the cytokines (182). Parallel increases in IL-6 and IL-10 were observed in patients with obsessive-compulsive disorder (183) and chronic hepatitis where the correlation between levels of these proteins was particularly high (*P* = 0.005) (184). Those changes were not accompanied by any change in IFN-γ levels, perhaps indicating a critical role of IDO metabolites and the IL-6:IL-10 balance in the regulation of IFN-γ. It may be relevant that expression of the IL-10 receptor is also affected by tryptophan catabolites, since it is increased by IFN-γ stimulation and activation of AHRs by kynurenine regulates activity of the promoter region of the IL-10 receptor α-subunit to increase receptor expression (185).

Increasing evidence indicates that IDO activation and IL-10 production can be induced by the same stimuli, leading to the view that the anti-inflammatory and tolerogenic actions of both proteins may be at least complementary and potentially synergistic (186, 187). Functionally, IDO and IL-10 show important interactions with some degree of mutual redundancy. Thus, amniotic fluid stem cells which are closely related to mesenchymal stem cells and possess the same profile of molecular markers, powerfully suppress the proliferation of peripheral blood mononuclear cells induced by phorbol-12-myristate-13-acetate (PMA). This inhibition is mediated by the combination of IDO and IL-10 production by the amniotic cells, with both being required for maximal suppression of proliferation (188).

The relationship between IDO and IL-10 may be particularly relevant in the presence of microbial invasion since some bacteria can induce DCs to facilitate IL-10 production by subpopulations of T cells such as the FoxP3-negative Tr1-like cells. This is achieved by the microbial induction of appropriate T cell polarizing molecules including IDO1 (189). Consistent with the overall anti-inflammatory balance which this generates, the production of IDO1 and IL-10 is accompanied by a reduction in the secretion of pro-inflammatory TNF-α and IL-12 components. The simultaneous presence of IDO1 and IL-10 can also influence non-infective inflammation such as that associated with tumor development. For many tumors, it is recognized that compounds with characteristics of Damage-Associated Molecular Patterns in the local microenvironment promote tolerogenic dendritic and mesenchymal stem cells which in turn induce the stable co-expression of IDO and IL-10 to further suppress lymphocyte aggression (190). Among the compounds able to initiate this activity are uric acid and the S100A4 protein, both of which have been linked to a variety of inflammatory states in peripheral and central tissues.

## Environmental Factors and the Kynurenine Pathway: Development and Epigenetics

It is a common experience that the existence and severity of arthritic symptoms can vary substantially throughout life, at different times of day, with changes in affective status (mood, stress, anxiety etc.) or with changes in dietary habits. It is important to recognize, therefore, that many such factors do impact directly on the kynurenine pathway and could modulate inflammatory symptoms, peripheral, or central. Exposure to stressful conditions activates the hypothalamo-pituitary-adrenal axis leading to the secretion of corticosteroids which are potent inducers of TDO and will therefore increase kynurenine pathway activity. Cruciferous vegetables in particular contain alkaloids such as brassinins which inhibit IDO1 (191) and a variety of indole-derived compounds such as indole-3-carbinol and di-indolyl-methane which are agonists at AHRs (192, 193), thus regulating the IDO/TDO-kynurenine-AHR feedback cycle described above and giving the compounds significant anti-inflammatory properties (194, 195). The influence of such factors may be of paramount importance in determining the occurrence and severity of arthritis and related disorders, since we have demonstrated a role of the kynurenine pathway in tissue development of the embryo, and the methylation state of IDO1 appears to determine the magnitude of induced arthritis.

### Embryonic Development

The normal, physiological roles of the kynurenine pathway have received less attention than their potential pathological relevance but recent reports have indicated the probability of important functions in early development of the embryo. The treatment of pregnant rats in late gestation with an inhibitor of KMO (Figure 1), results in an accumulation of kynurenine as well as promoting its transamination to kynurenic acid. These changes resulted in significant molecular, structural, immunocytochemical and functional (electrophysiological) changes in neonates produced by the treated dams and changes in all these parameters persisted into adulthood (161–164). Similar results were obtained by the administration of kynurenine itself to pregnant animals (196–199), and in animals lacking KMO by genetic manipulation (165, 166).

These results indicate that the kynurenine pathway is playing a significant role in early development and the initial hypothesis to explain these effects was based on the known importance of glutamate and its receptors in the early formation of the brain. In particular, the NMDA-sensitive subtype of receptors are involved in neurogenesis, progenitor cell migration, axon and dendrite growth and guidance as well as spine and synapse formation. The activity of these NMDA receptors—and therefore brain development—would be dependent on the ratio of the NMDA receptor agonist quinolinic acid and the antagonist kynurenic acid. The occurrence and long-term maintenance of altered brain structure and function might contribute to the development of neurological and psychiatric disorders in adult life. The most established examples of such “neurodevelopmental” disorders are schizophrenia (145–147, 197, 199, 200) and major depression (154, 155) including suicide vulnerability (135).

If these changes in CNS development are mirrored in the actions of kynurenines on the immune system, they could contribute significantly to immune system responses and to the susceptibility of offspring to a range of immunological problems including autoimmune diseases such as RA. Thus, in the brain development studies there may have been changes in immune function mediated by the altered levels of kynurenines, such as abnormal cytokine or chemokine levels in the pregnant dam or embryonic brain, changes to microglial activation, or modifications to peripheral leukocyte function and their infiltration into the pre- or postnatal brain. Any effects of prenatal interference with the kynurenine pathway on immune function would represent a highly important area of investigation, especially in the light of evidence that some leukocyte populations express NMDA receptors and other targets of kynurenine and its catabolites such as AHRs and the G-protein coupled receptor GPR35 (82).

While there is a growing literature on the effects of prenatal inflammatory stimuli on CNS development and function in the adult offspring (161–164, 201–204), few studies have yet addressed the immunological consequences of such maternal factors. In one such study, however, it is clear that prenatal activation of the maternal immune system using bacterial LPS or the viral mimetic poly(inosinic:cytidylic) acid (poly[I:C], PIC) can affect the concentrations of several cytokines in the offspring in parallel with changes in expression of IDO (205). The most interesting result of this study was that a repeat immunological challenge in adulthood produced less change in the test animals than controls, indicating a long-lasting and possibly permanent depression of immune system function which could have significant implications for the development of autoimmune disease. However, a previous study reached the opposite conclusion, that prenatal immune activation induced increased adaptive immune responses in the offspring (206) raising the possibility that subtle differences in experimental animals or procedures may have a major influence on the outcome.

While these factors do not fall under the classification of “epigenetic” they will clearly interact with epigenetic processes described below in determining the final, overall activity and effectiveness of the kynurenine pathway in neurological and immunological function in postnatal life.

### Epigenetics of IDO

If the kynurenine pathway is as widely and fundamentally important as the above discussions imply, any modifications to the various components of the pathway would carry substantial implications for a variety of disorders. Relevant changes could include genetic mutations or epigenetic changes. The latter do not involve changes directly to the genetic machinery or nucleotide sequence, but consist of minor changes to the gene or related segments of the chromosome such as promoter sequences, which alter the functionality of that gene. This may change the extent to which a section of gene is transcribed, or the properties of the transcribed protein. Epigenetic changes are often the result of environmental factors (diet, stress, disease) which affect the activity of key enzymes such as acetyl- or methyl- donors, altering their activity on the genome. Epigenetic changes are often reversible but, if they affect the germ-line cells, they may be inherited.

Several of the disorders discussed above may be susceptible to epigenetic influences. The brains of patients with Huntington's disease exhibit evidence of alterations in methylation status (207, 208) or acetylation status (209) and inhibition of a histone deacetylase may prevent the development of cognitive deficits as well as huntingtin expansion (210). Such changes may contribute significantly to the course of the disorder and its heritability, especially since DNA methylation has been shown to produce extension of the mutant CAG repeat sequence (211).

RA may involve defects in acetylation (212), reflected in the beneficial activity of a histone deacetylase inhibitor in CIA (213). It is also likely that there are changes in DNA methylation (214–217) as has been noted in regulatory T cells (218) and synovial fibroblasts (219). The methylation pattern has been claimed to reflect therapeutic efficacy of the TNF-α inhibitor etanercept (220) and so may be relevant to explaining the non-responsiveness to this drug of some patients.

The aberrant methylation of Treg cells, affecting the *FOXP3* and *CTLA4* genes, reduces their immunosuppressant activity. We have found that the DNA-demethylating compound decitabine reduces this suppression and restores immunosuppression associated with increased expression of Treg markers. In the CIA model of arthritis decitabine increased the suppression function of Treg cells along with a decrease in pro-inflammatory Th1 and Th17 cells and their infiltration into arthritic paws. Of major relevance to the kynurenine pathway, these effects of demethylation were associated with increased expression of IDO1 which is normally an important aspect of the immunosuppressant behavior of Treg cells mediated by the CTLA-4 ligation of B7 proteins, and further differentiation of Treg cells by the promotion of FoxP3 expression.

When CIA was induced in IDO1-deficient mice on a C57/BL6N.Q (H-2^q^) background), decitabine administration reduced both the early symptoms and pathology of the disorder but also reduced the expression of transcription factors characterizing pro-inflammatory cells (IFNγ+ and Tbet^+^ in Th1; IL17+ and RoRγt^+^ in Th17 cells). In contrast, symptoms were exacerbated in the later stages of disease in parallel with a loss of FoxP3^+^ Tregs and an increased number of Tbet^+^ Th1 and RoRγt^+^ Th17 cells. This time course would be consistent with the concept that IDO1 activity is a critical feature of the interactions needed to maintain long-term Treg-mediated immune tolerance. Decitabine also increased the number of IDO1-positive monocytes while a combination of ADC/decitabine and IFN-γ allowed myeloid DCs to increase their IDO1 expression.

Overall, because of its critical, central role in immune function and tolerogenesis, the methylation of IDO1 appears to be an important factor in determining its activity. If these factors affect germline DNA, as noted above, they may significantly affect the immunological competence of offspring and their susceptibility to a range of disorders.

## Clinical Potential

This review has introduced a few of the many disorders afflicting peripheral tissues or the CNS in which inflammation is implicated, but that is enough to recognize the therapeutic potential of influencing the kynurenine pathway by pharmacological interference. Many academic and commercial laboratories have demonstrated the promise of analogs or derivatives of kynurenine and its catabolites to act as receptor agonists, antagonists or enzyme inhibitors (138, 221, 222).

In the CNS work has been concentrated on inhibitors of KMO to reduce quinolinic acid synthesis and thereby reduce neural activity and excitotoxicity in neurodegenerative disorders as well as in the suppression of peripheral inflammation, especially in the pancreas (223–226). A different approach is in the development of KAT inhibitors intended to reduce kynurenic acid formation in psychiatric disorders such as schizophrenia (227–230). Of course these two approaches, being essentially contrary in their objectives, raise concerns that schizoid symptoms might be induced in response to KMO inhibition, or that KAT inhibition could divert more kynurenine via KMO to quinolinic acid.

In a similar vein, there has been a major effort to develop inhibitors of IDO1 to prevent the immune-suppressant activity of this enzyme and thus to drive tumor cell death or to facilitate the effects of anti-tumor drugs (231–235). In principle, this approach might lead to the initiation or exacerbation of clinical inflammatory and autoimmune disorders described above. While this would remain a problem which would need careful monitoring, clinical trials with IDO1 inhibitors have recently been found to be less effective anti-cancer agents than anticipated (111–113), raising doubts about the continuation of this strategy.

Despite these concerns, major advances are being made in kynurenine-related treatments for Huntington's disease and schizophrenia, and the range of conditions potentially amenable to kynurenine-related therapy continues to escalate. The factors which can recruit kynurenine pathway involvement such as infection, inflammation, dietary changes, various forms of stress and others make it highly likely that the kynurenine pathway will prove to be a valuable source of new therapeutic agents in the near future.

## Summary and Conclusions

Some of the inter-relationships between IDO or its kynurenine-derived catabolites and aspects of the immune system have been discussed, focusing on several examples of disorders affecting peripheral tissues or the CNS. In many cases there are important questions to be resolved, such as which components of the kynurenine pathway are responsible for different elements of immune regulation. It seems likely that a fuller appreciation of these issues will not only help to understand the molecular basis of some disorders, but will further the development of increasingly sophisticated and targeted therapies (236). This will be especially important if methods can be identified to modify or prevent epigenetic changes which alter the expression or functional capacity of relevant enzymes, receptors or transduction systems. Finally, more detailed investigation of the immunological consequences of stress, infection and immune system activation during pregnancy—all of which involve activity in the kynurenine pathway—may be highly relevant to understanding postnatal susceptibility to autoimmune disorders and cancer, much as recent work has shown lasting effects on the nervous system. With growing interest in the importance of neuroimmune interactions for disease development and resolution, a combined knowledge of both these areas might yield synergistic advances in medicine and therapeutics.

## Author Contributions

All authors contributed to the writing and proof-reading of this manuscript.

### Conflict of Interest

The authors declare that the research was conducted in the absence of any commercial or financial relationships that could be construed as a potential conflict of interest.
